# The effects of switching from etelcalcetide to upacicalcet in hemodialysis patients with secondary hyperparathyroidism 

**DOI:** 10.5414/CN111695

**Published:** 2025-08-20

**Authors:** Eiichi Sato, Miyako Urata, Shohei Sato, Takao Ono, Manaka Degawa, Hongmei Lu, Mayumi Nomura, Daisuke Matsumura, Noriaki Moriyama, Mayuko Amaha, Tsukasa Nakamura

**Affiliations:** 1 Division of Nephrology, Department of Internal Medicine, Shinmatsudo Central General Hospital, Matsudo, and; 2 Department of Nephrology, Kashiwa Forest Clinic, Kashiwa, Japan

**Keywords:** secondary hyperparathyroidism, etelcalcetide, upacicalcet, activated vitamin D analogs

## Abstract

Aims: No English-language research papers have reported on the clinical use of upacicalcet, a novel intravenous calcimimetic agent for the treatment of secondary hyperparathyroidism (SHPT) in hemodialysis patients. Therefore, this study aimed to investigate the outcomes of switching from etelcalcetide to upacicalcet. Materials and methods: The subjects included 37 hemodialysis patients with SHPT treated with etelcalcetide before switching to upacicalcet. This study was a single-center retrospective study conducted in Japan. Serum levels of corrected calcium (Ca), phosphorus (P), intact parathyroid hormone (iPTH), and the dose of maxacalcitol were assessed at 3 and 6 months after switching to upacicalcet. Results: As a result of the switch from etelcalcetide to upacicalcet, the serum corrected Ca level remained unchanged, from 8.9 ± 0.6 to 9.1 ± 0.7 mg/dL (p = 0.104) at 3 months and to 9.0 ± 0.6 mg/dL (p = 0.197) at 6 months. Meanwhile, the serum P level decreased from 6.3 ± 1.5 to 5.8 ± 1.5 mg/dL (p = 0.069) at 3 months and to 5.9 ± 1.9 mg/dL (p = 0.039) at 6 months. The iPTH level increased slightly, from 153.8 ± 100.3 pg/mL to 176.4 ± 124.6 pg/mL (p = 0.337) at 3 months and to 206.5 ± 168.7 pg/mL (p = 0.017) at 6 months. Multiple regression analysis revealed that the change in iPTH was related to the change in P levels. Conclusion: These findings suggested that upacicalcet may be a useful option for managing serum P levels in hemodialysis patients with SHPT.

## Introduction 

The progression of chronic kidney disease leads to the development of secondary hyperparathyroidism (SHPT) as a complication. The symptoms of SHPT include bone disorders, vascular calcification, cardiovascular disorders, and calcific uremic arteriostenosis, and this leads to an increase in the incidence of cardiovascular disorders and the risk of death [1]. SHPT is caused by an abnormality in mineral metabolism [high phosphorus (P) level, low calcium (Ca) level, and vitamin D deficiency] due to the downregulation of the vitamin D receptor and calcium-sensing receptor (CaSR) in the parathyroid gland, which results in the excessive secretion of the parathyroid hormone (PTH) [2]. 

Treatment of SHPT involves correcting elevated P level, low Ca level, and high PTH in the serum. Combinations of activated vitamin D analogs and CaSR agonists (calcimimetics) are used to appropriately manage these parameters based on individual patients’ serum P and Ca levels. Activated vitamin D analogs promote the absorption of Ca and P in the intestinal tract while decreasing the synthesis of PTH [2] and increasing the serum Ca and P levels through reuptake in the kidneys [3]. Meanwhile, calcimimetics increase the sensitivity of CaSR in the parathyroid gland to suppress PTH, which consequentially decreases the serum levels of Ca and P [4]. The calcimimetics approved in Japan at present are cinacalcet hydrochloride (cinacalcet), evocalcet, etelcalcetide hydrochloride (etelcalcetide), and upacicalcet sodium hydrate (upacicalcet), a novel intravenous calcimimetic agent. For cinacalcet and evocalcet, the binding site is the transmembrane domain, and the metabolic organ is the liver, whereas for etelcalcetide and upacicalcet, the binding site is the extracellular domain, and the main excretion route is removal by dialysis. Of these, etelcalcetide has been approved in many countries as the first intravenous calcimimetic agent for use in SHPT [5]. Intravenous calcimimetics can be administered to patients who have concerns about treatment adherence, without the burden of drug administration. Meanwhile, a comparative study of etelcalcetide and cinacalcet revealed that etelcalcetide was associated with a greater decrease in Ca levels [6]. Also, Palmer et al. [7] reported that hypocalcemia appeared to occur more frequently for etelcalcetide than cinacalcet and evocalcet. A recent 9-year prospective cohort study of patients prescribed cinacalcet reported that hypocalcemia was associated with sudden death as well as death from hemorrhagic stroke, heart failure, and ischemic heart disease [8]. This suggests that calcimimetics with a lower risk of hypocalcemia are preferable. In an in vitro study by Goto et al. [9], upacicalcet was not found to activate CaSR in the absence of extracellular Ca ions, whereas etelcalcetide was reported to activate CaSR under the same conditions. Additionally, an in vivo study showed that serum Ca levels decreased only slightly after administration of upacicalcet at the effective dose or higher, whereas etelcalcetide caused a further decrease in serum Ca at the effective dose or higher [9]. These findings suggest that upacicalcet may pose a lower risk of hypocalcemia compared to other calcimimetics. 

No English-language research papers have reported on the use of upacicalcet in real-world clinical practice or data on switching from etelcalcetide to upacicalcet. Etelcalcetide is ~ 73% covalently bound to serum albumin after administration, and only ~ 60 – 70% of the unchanged form (~ 17%) is removed by hemodialysis. After dialysis, the unchanged form increases in blood concentration as it is converted from etelcalcetide covalently bound to serum albumin, leading to an increase in blood concentration with repeated dosing. When [14C]-labeled etelcalcetide is administered intravenously, 59.6% is excreted into dialysate within 175 days. On the other hand, up to 90% of upacicalcet is present in the unchanged form after administration and does not covalently bind to albumin, so 78.4 – 100% of the unchanged form is removed by hemodialysis. Therefore, there are differences in accumulation between the two drugs [10, 11, 12], and this study aimed to assess the effects of switching from etelcalcetide to upacicalcet on serum levels of corrected Ca, P, intact PTH (iPTH) in real-world clinical practice, as well as the dose of maxacalcitol, and to identify factors influencing changes in iPTH levels. 

## Materials and methods 

### Study design 

This study was a single-center retrospective study conducted in Japan. Only existing data were included in the analysis, and no new data were collected. The opt-out policy was adopted for the use of the existing data. Following the review and approval by the Clinical Research Ethical Review Board of Shido, Inc. (approved May 23, 2024, approval number: S20240315-1), the study was registered in the Clinical Trial Registry (https://www.umin.ac.jp/; UMIN000054865) and was conducted according to the ethical principles set out in the Declaration of Helsinki and Ethical Guidelines for Medical and Biological Research Involving Human Subjects. The opt-out document was presented internally at the study site, and the opt-out period was provided. If participants refused the use of their data during the opt-out period or between the end of the opt-out period and the completion of the analysis, the relevant data were deleted from the study records. Patients who met the eligibility criteria were identified through their medical records, and the data were then extracted and analyzed. The study period (period for extraction of information) was from May 2022 to January 2023. 

### Subjects 

**Inclusion criteria**

Patients who met all of the following criteria were included in this study:

- Hemodialysis patients with SHPT who used etelcalcetide from May 2022 to June 2022 and who switched to upacicalcet.
- Patients who continued upacicalcet for 6 months after switching to upacicalcet and for whom the blood test values at 6 months had been obtained.

No washout period was provided for switching from etelcalcetide to upacicalcet. 

**Exclusion criteria**

Patients who met any of the following criteria were excluded from this study:

- Patients who discontinued treatment with upacicalcet.
- Patients who refused the use of data through the opt-out policy.

### Endpoints 

The endpoints were set as the serum levels of corrected Ca, P, iPTH at the time of switching from etelcalcetide to upacicalcet, and at 3 and 6 months after switching, as well as the mean dose of maxacalcitol (μg/week) before switching, at 2 – 4 months after switching, and 4 – 6 months after switching. An analysis was also conducted on the factors relating to the change in iPTH at 6 months after switching. 

### Statistical analyses 

Patients who met all of the inclusion criteria and none of the exclusion criteria were included in the analysis population. The paired t-test and Wilcoxon signed rank test were used for the test on the comparison of values from the time of switching to upacicalcet and at 3 and 6 months after switching with a two-sided confidence level of 5%. We analyzed the distribution of each data set and used paired t-test for Ca and P, and Wilcoxon signed rank test for iPTH. Multiplicity was not adjusted. The analysis result was displayed as the mean ± standard deviation for continuous variables and number of patients (percentage) for the nominal scale. A multiple regression analysis was used for the factor analysis on the change in iPTH at 6 months after switching. An analysis was performed using Excel Statistics Ver. 7.0. 

## Results 

**Figure 1:**
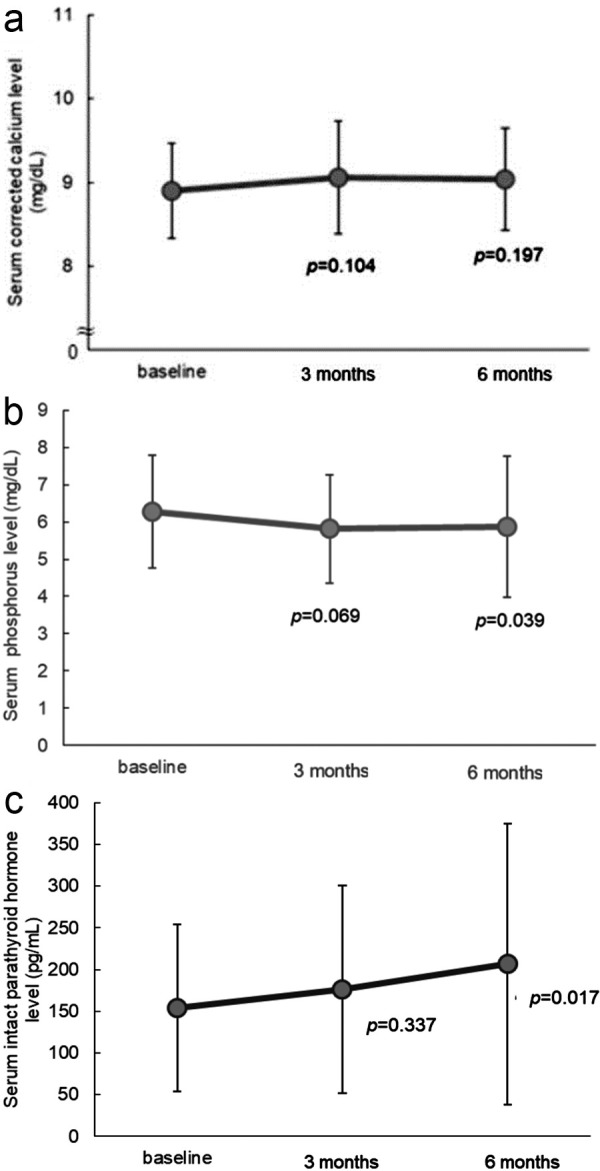
Time course of serum corrected calcium level (a), serum phosphorus level (b), and serum intact parathyroid hormone level (c). Data are presented as mean ± SD.

**Figure 2:**
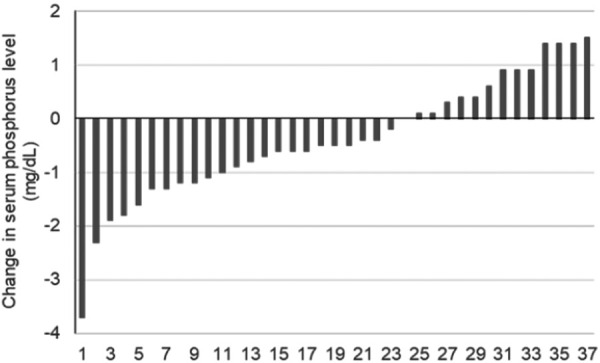
Changes in phosphorus level for individual patients from baseline to week 24.

**Figure 3:**
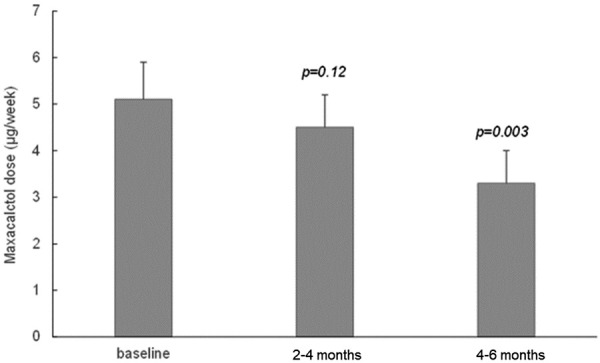
Time course of the dose of maxacalcitol (25 patients). Data are presented as mean ± SD.

The study included 28 males (75.7%) and 9 females (24.3%) with a mean age of 64.3 ± 2.3 years and a mean dialysis history of 86 ± 11 months. Primary disease was diabetic nephropathy in 18 patients (48.6%), nephrosclerosis in 12 patients (32.4%), IgA nephropathy in 2 patients (5.4%), membranous nephropathy in 1 patient (2.7%), and not specified in 4 patients (10.8%) (Table 1). The dose was switched to about upacicalcet 75 μg/week if the dose of etelcalcetide was 7.5 mg/week. At the time of switching from etelcalcetide to upacicalcet and at 3 and 6 months after switching, the serum corrected Ca level changed from 8.9 ± 0.6 mg/dL to 9.1 ± 0.7 mg/dL (p = 0.104) and 9.0 ± 0.6 mg/dL (p = 0.197), respectively (Figure 1a). The serum P level changed from 6.3 ± 1.5 mg/dL to 5.8 ± 1.5 mg/dL (p = 0.069) and 5.9 ± 1.9 mg/dL (p = 0.039) (Figure 1b). The iPTH level changed from 153.8 ± 100.3 pg/mL to 176.4 ± 124.6 pg/mL (p = 0.337) and 206.5 ± 168.7 pg/mL (p = 0.017) (Figure 1c). No significant change was observed in the serum corrected Ca level, whereas the serum P level after 6 months showed a significant decrease, and the iPTH level after 6 months showed a significant increase. The change in the serum P level after switching ranged from –3.7 to 1.5 mg/dL, with 23 patients (62.2%) showing a decrease and 13 patients (35.1%) showing an increase (Figure 2). The dose of etelcalcetide before switching was 7.5 ± 0.8 mg/week, while the dose of upacicalcet at the time of switching was 75.7 ± 6.8 μg/week, increasing to 96.3 ± 14.2 μg/week at 6 months. The dose of maxacalcitol decreased significantly from 5.1 ± 0.8 μg/week before the switch to upacicalcet to 3.3 ± 0.7 μg/week (p = 0.003) at 4 – 6 months after the switch (Figure 3). Moreover, no major change was observed in P adsorbents (Supplemental Table 1). The result of the factor analysis on the change in the serum iPTH level after 6 months showed a relationship with a significance only in the change in the serum P level from baseline to 6 months (Table 2). 

## Discussion 

This study is the first English-language report on switching treatment from etelcalcetide to upacicalcet in clinical practice. The results showed that the switch did not significantly affect the serum corrected Ca level. While an increase in iPTH was observed, a significant decrease in the serum P levels was noted. 

### Significant decrease in serum P level 

According to the statement in the international guideline Kidney Disease: Improving Global Outcomes (KDIGO) 2017 [13], the P level should be decreased to the normal range if it is high. Isaka et al. [14] reported that reducing the serum P level in dialysis patients to the normal range can help improve the progression of coronary calcification, a key predictor of the all-cause mortality in patients undergoing dialysis. Additionally, Goto et al. [15] reported that in hyperphosphatemic hemodialysis patients, a decrease in serum phosphorus levels was associated with a reduction in mortality risk. Specifically, in patients with a history of atherosclerotic cardiovascular disease (CVD) or diabetic nephropathy, a positive linear relationship was observed between serum phosphorus levels and CVD mortality rates [15, 16]. Although the serum P level decreased significantly after switching treatment from etelcalcetide to upacicalcet, it is believed that a reduction in the dose of maxacalcitol contributed to this change. 

Several reports are available on the relationship between activated vitamin D analogs and P. According to Akizawa et al. [17], 12-week administration of maxacalcitol in 203 patients with SHPT has resulted in a tendency for a dose-dependent increase in Ca and P, which was thought to require caution. Also, Moe and Drücke [1] reported that an activated vitamin D analog could increase the absorption of Ca and P in the intestinal tract to trigger excessive suppression of PTH, which then decreases the number of active osteoblasts and thereby directly decreases bone formation [1, 18]. Furthermore, Cummingham et al. [2] reported that a high dose of an activated vitamin D analog causes hypercalcemia and hyperphosphatemia and is related to ectopic calcification. As a result of switching from etelcalcetide to upacicalcet, the serum P levels decreased from 6.3 ± 1.5 to 5.8 ± 1.5 mg/dL. This improvement of ~ 0.5 mg/dL in P is very important because it can reduce the risk of calcification and bone disorders and may impact patient survival. Although the fundamental reason for the decrease in serum P levels is not entirely clear, the reduction in vitamin D dosage was consistent with the observed decrease in serum phosphorus levels. 

### Effect on Ca 

In this study, the dose of an activated vitamin D analog was decreased after switching the treatment from etelcalcetide to upacicalcet; however, no significant change was observed for the serum corrected Ca level. This finding suggests that upacicalcet has a weak effect in decreasing Ca. Goto et al. [9] reported that while etelcalcetide exhibits agonist activity in the absence of extracellular Ca and decreases serum Ca levels in a dose-dependent manner, upacicalcet activates CaSR in a manner dependent on extracellular Ca levels, without exhibiting agonist activity at or below the physiological Ca level. This difference was thought to contribute to the lower risk of hypocalcemia associated with upacicalcet compared to etelcalcetide. Moreover, upacicalcet is available in seven formulations ranging from 25 to 300 μg. Since 78.4 – 100% is removed by hemodialysis, it is considered a drug that facilitates clinical dose adjustment while monitoring patients’ Ca levels, compared to etelcalcetide. This characteristic is also believed to be a factor in reducing the risk of hypocalcemia. Sato et al. [19] reported that the action of upacicalcet is mediated by the amino acid binding site of CaSR unlike the conventional calcimimetics. It has also been reported that interactions with multiple amino acid binding residues transition CaSR from an inactive state to an intermediate activation state, and in the presence of Ca2+, activate CaSR [20]. Calcimimetics have been reported to act not only on CaSR in the parathyroid gland but also on CaSR in the intestinal tract and bones [21, 22]. Upacicalcet may have a different degree of action on these CaSR compared to etelcalcetide. No significant change in serum Ca levels was observed in this study. Furthermore, it was reported that the incidence of severe hypocalcemia was 0 – 2% with upacicalcet and 5% with etelcalcetide [6, 12, 23]. Upacicalcet may be less likely to cause hypocalcemia than etelcalcetide and may also reduce complications associated with hypocalcemia. A comparative study should be conducted to further investigate this finding. 

### Equivalent dose of upacicalcet and etelcalcetide and effect of PTH 

In this study, the iPTH level increased significantly after switching from etelcalcetide 7.5 mg/week to upacicalcet 75 μg/week; however, this remained within the target range of 60 – 240 pg/mL, as set by the Japanese Society for Dialysis Therapy [24]. The mean dose of upacicalcet 6 months after switching was ~ 100 μg/week, suggesting that the effect of the decrease in iPTH for etelcalcetide 7.5 mg /week was equivalent to that of upacicalcet 100 μg/week or higher dose. We conducted a factor analysis with the change in the iPTH level as the target variable and found a significant relationship with the change in the P level. Based on this, the increase in the iPTH level was likely influenced by the increase in the P level observed in some patients (Table 2) (Figure 2). Furthermore, since the clinical dose of upacicalcet ranges from 25 to 300 μg/session, and the mean dose at 6 months was ~ 100 μg/week, there is potential for further dose escalation. Considering these factors, the increase in iPTH levels is likely due to the increase in P levels in some patients and the insufficient dose increase for upacicalcet. 

### Limitations 

This report presents a single-center retrospective study on the switch from etelcalcetide to upacicalcet, excluding patients who withdrew before 6 months or transferred to another hospital. Factors such as selection bias and the impact of unmeasured and unadjusted confounding factors should be considered when interpreting the results. As the study was retrospective without deliberate intervention, it only evaluated the switch from etelcalcetide to upacicalcet, not vice versa. There was no washout period between the switch from etelcalcetide to upacicalcet as this study examines the switch in real-world settings where washout is not common practice. Confounding factors such as comorbidities and treatment history were not included in the multivariate analysis. The study did not perform sample size calculations and had only 37 patients, so there are limitations to generalizing these results. Also, we did not consider multiplicity, increasing the risk of false positives. To validate the results of this study, it is necessary to attempt a direct comparison randomized trial or conduct a crossover trial. 

## Conclusion 

In the treatment of SHPT in hemodialysis patients, the serum-corrected Ca level remained unchanged 6 months after switching from etelcalcetide to upacicalcet; however, a decrease in serum P levels and an increase in iPTH levels were observed. The reduction in the dose of maxacalcitol is thought to have contributed to the decrease in serum P levels. Switching from etelcalcetide to upacicalcet was considered to be a useful option for hemodialysis patients with SHPT particularly from the perspective of P control. 

## Acknowledgment 

We would like to thank GROVA (www.glova.co.jp/) for the English translation. 

## Data availability 

The biochemical data used to support the findings of this study belong to the study team. For confidentiality reasons, the datasets are not publicly available. However, the datasets can be obtained from the corresponding author with permission from the study team upon reasonable request. 

## Authors’ contributions 

Conceived and designed the experiments: ES. Enrolled patients: ES, MU, SS, TO, MD, HL, MN, DM, NM, MA, and TN. Analyzed the data/interpretation: ES, MU, SS, TO, MD, HL, MN, DM, NM, MA, and TN. Wrote the first draft of the manuscript: ES. Contributed to the writing of the manuscript: ES. All authors agreed with the manuscript’s results and conclusions. All authors have read and confirm that they meet ICMJE criteria for authorship. 

## Funding 

This study was conducted with funds provided by Sanwa Kagaku Kenkyusho Co., Ltd., according to the contract. 

## Conflict of interest 

This study was conducted with funds provided by Sanwa Kagaku Kenkyusho Co., Ltd., according to the contract; however, this did not have an impact on the conduct or evaluation of this study. In addition, funds were not provided to the study facility or the investigators. Sanwa Kagaku Kenkyusho Co., Ltd., only provided funds for the outsourcing of the operations. 

**Table 1.** Participant characteristics (n = 37).

Sex	Male: 28 (75.7%), female: 9 (24.3%)
Age	64.3 ± 2.3 years
Dialysis history	86 ± 11 months
Primary disease Diabetic nephropathy Nephrosclerosis IgA nephropathy Membranous nephropathy Not specified	18 (48.6%) 12 (32.4%) 2 (5.4%) 1 (2.7%) 4 (10.8%)
Concentration of Ca in dialysate	3.0 mEq/L: 37 (100%)

Data are presented as number (percent) or mean ± SD. Ca = calcium.

**Table 2.** Factor analysis on the change in iPTH.

Variable	Partial regression coefficient	95% CI (Lower limit)	95% CI (Upper limit)	p-value
Ca at the start (mg/dL)	46.2	–18.7	111.0	0.156
P at the start (mg/dL)	2.4	–23.0	27.7	0.849
Change in P (P level at 6 months – P level at baseline) (mg/dL)	40.1	3.8	76.4	0.032
iPTH at the start (pg/mL)	0.1	–0.2	0.5	0.470
Sex (male: 1, female: 0)	2.0	–85.1	89.0	0.963
Age (years)	–1.0	–3.8	1.9	0.496
Dose of upacicalcet immediately after switching (μg/week)	–0.8	–1.7	0.2	0.133
Whether the dose of upacicalcet is increased (increase: 1, not: 0)	–11.6	–89.2	66.1	0.762
Dose of maxacalcitol immediately after switching (μg/week)	–6.2	–16.2	3.8	0.211

Ca = corrected calcium; P = phosphorus; iPTH = intact parathyroid hormone; 95% CI: 95% confidence interval.

## Supplemental material

Supplemental material Dose of phosphorus adsorbent
