# Supplementary material for: The effects of switching from etelcalcetide to upacicalcet in hemodialysis patients with secondary hyperparathyroidism
Source: Clin Nephrol. 2025 Aug 20;104(5):310–7. doi: 10.5414/CN111695 (PMC12573280; doi:10.5414/CN111695)
Supplement: Supplemental material [file clinnephrol-104-310-S01.pdf]

Supp. 1. Dose of phosphorus adsorbent

	At switching			After 3 months			After 6 months		
	n	mean	SD	n	mean	SD	n	mean	SD
Sucroferric oxyhydroxide (mg/day)	7	1214	509	7	1107	518	7	1107	518
Ferric citrate hydrate (mg/day)	13	1558	647	13	1558	647	13	1558	647
Precipitated calcium carbonate (mg/day)	16	2250	949	16	2250	949	16	2344	944
Bixalomer (mg/day)	3	833	629	3	833	629	3	833	629
Lanthanum carbonate hydrate (mg/day)	5	1500	0	6	1500	0	7	1393	283
